# Effects of seedling-stage LED supplementary lighting on the eating quality and textural properties of oriental melon (*Cucumis melo* L. var. *makuwa* Makino) fruits at maturity

**DOI:** 10.3389/fpls.2026.1818870

**Published:** 2026-04-15

**Authors:** Ximing Xu, Jieyu Wu, Yufeng Chen, Mingying Shi, Ziyu Chen, Yuquan Lin, Bingliang Wang, Hongxia Ye, Xingren Shi

**Affiliations:** 1 The Key Laboratory for Quality Improvement of Agricultural Products of Zhejiang Province, College of Advanced Agricultural Sciences, Zhejiang A&F University, Hangzhou, China; 2 Huzhou Wuxing Jinnong Ecological Agriculture Development Co., Ltd., Huzhou, China; 3 State Key Laboratory for Managing Biotic and Chemical Threats to the Quality and Safety of Agro-Products, Institute of Plant Virology, Ningbo University, Ningbo, China; 4 Institute of Vegetable Science, Zhejiang University, Hangzhou, China

**Keywords:** eating quality, LED supplementary lighting, oriental melon, seedling stage, sensory evaluation, texture

## Abstract

**Introduction:**

Low-light stress during winter compromises the sensory quality of off-season oriental melons (*Cucumis melo* L. var. *makuwa* Makino) in cultivation. Although LED supplemental lighting is commonly used to alleviate low-light stress in horticulture, its long-term programming effects on fruit quality remain largely unknown.

**Methods:**

This study investigated whether early light signals from different LED spectra (red:blue ratios of 1:1 and 5:1) and intensities (full‑spectrum white at 18, 48, and 60 W) could persistently shape the texture and eating quality of mature fruits in two cultivars, ‘Green Gem’ and ‘Young White Lady’. Seedling-stage lighting treatments were applied, and subsequent fruit quality parameters—including perceived sweetness, fruit firmness, total soluble solids (TSS), titratable acidity (TA), and sugar-acid ratio—were measured.

**Results:**

Key cultivar‑specific programming effects were identified. For ‘Green Gem’, seedling‑stage lighting with R:B = 5:1 at 18 W most effectively enhanced perceived sweetness and fruit firmness. For ‘Young White Lady’, full‑spectrum light at 48 W optimally boosted TSS (11.9 ± 1.5 °Brix), while 60 W reduced TA, yielding a superior sugar‑acid ratio (95.5 ± 7.0). Additionally, a high R:B ratio strongly correlated with increased acidity (ρ = 0.579).

**Discussion:**

These findings reveal a trade‑off between flavor and texture, as high R:B ratios promote acidity but may affect other quality attributes. Seedling‑stage spectral management offers an energy‑efficient strategy for precision quality control in protected horticulture.

## 1 Introduction

Oriental melon (*Cucumis melo* L. var. *makuwa* Makino) is a high-value fruit in East Asia, prized for its sweet taste and crisp texture (Kim et al., 2016; Shin et al., 2019; de Freitas et al., 2024). The fruit’s sweetness is primarily derived from its soluble sugar content, while its texture is a critical quality parameter often evaluated through sensory and instrumental methods (Farcuh et al., 2020; Pan et al., 2022; Tzuri et al., 2025; Bianchi et al., 2016; Lázaro and de Lorenzo, 2015). However, the off-season protected cultivation essential for year-round supply is frequently compromised by low-light stress during winter and early spring (Yamaguchi et al., 1977; Hossain et al., 2025; Wei et al., 2020; Li et al., 2016). This abiotic stress directly impairs the qualities that define the fruit’s value: it weakens sink strength and represses sucrose metabolism, leading to significant reductions in fruit size, soluble sugars, amino acids, and textural properties (Yang et al., 2019; Gao et al., 2023), thereby decreasing the market value of oriental melon. Therefore, developing effective strategies to mitigate low-light limitations is crucial for sustaining both commercial yield and the prized quality of oriental melon.

Light is a fundamental environmental factor that regulates plant growth and development (Brini et al., 2022). Plants undergo morphological and physiological adaptations in response to their light environment. As key attributes of the light environment, photoperiod, light intensity, and light spectrum profoundly influence plant growth, development, nutrient accumulation, and quality formation (Li et al., 2021; Mei et al., 2023). Artificial supplementary lighting, particularly through the precise control afforded by Light Emitting Diode technology (LED) has emerged as an effective strategy to mitigate low-light stress and enhance the quality of fruit and vegetable crops in facility agriculture (Yan et al., 2018; Hossain et al., 2025; Mei et al., 2023; Chen et al., 2024). Solar radiation spans a spectral range primarily between 300 nm and 2600 nm, yet it is the photosynthetically active radiation (PAR) within 400–700 nm that directly drives photosynthetic processes (Moreno-Cuenca et al., 2025). Light supplies the energy required for photosynthesis and serves as an environmental signal that conveys critical external information. Light regulates plants through three key dimensions: spectrum, intensity, and photoperiod (Zhao et al., 2024). Among these wavelengths, red and blue light exert the most pronounced influence on plant physiology. Red light, which is most strongly absorbed by photosynthetic pigments in the range of 642–663 nm, demonstrates the highest absorption efficiency within the visible spectrum. Red light is maximally absorbed, driving the accumulation of soluble sugars and starch while orchestrating key physiological and biochemical processes to enhance photosynthesis (Chen et al., 2019). The high proportion of red light promotes an increase in plant height, stem thickness, and leaf area (Zhao et al., 2024). Red light supplementary lighting significantly bolstered melon seedling resistance to powdery mildew by elevating NADPH oxidase activity and promoting H_2_O_2_ accumulation (Wang et al., 2023). Similarly, blue light—exhibiting peak absorption at 430–453 nm is essential for chloroplast development, significantly increases chlorophyll and nitrogen content, and facilitates stomatal opening. These combined effects synergistically elevate photosynthetic efficiency in seedlings (Miao et al., 2019). However, it remains unknown whether this early enhancement in photosynthesis translates into a lasting programming effect that shapes fruit quality at maturity. Investigating this carry-over effect is the central objective of the present study.

Well-developed melon seedlings, cultivated under optimal light conditions, are critical for subsequent stress tolerance and plant growth (Zhao et al., 2024). However, the specific effects of seedling-stage supplemental lighting on final fruit taste quality, particularly under varied light intensity and spectral combinations, are not well understood. Although it is well documented that specific light spectra and intensities regulate photomorphogenesis, photosynthesis, and secondary metabolism in melon seedlings (Yang et al., 2019; Gao et al., 2023; Zhao et al., 2024), research to date has centered primarily on these short-term phenotypic effects, with limited attention to the immediate physiological responses during the treatment period itself. Consequently, whether the light signals perceived exclusively during this brief, early developmental window can exert a persistent, programming effect on fruit quality at maturity remains a critical and unexplored question. Addressing this knowledge gap is not only of scientific interest for understanding plant developmental memory but also of practical importance for developing energy-efficient cultivation strategies. Therefore, this study was designed to test the hypothesis that specific LED spectra and intensities applied solely during the seedling stage can induce carry-over effects that shape the sensory, nutritional, and textural quality of mature fruits in two oriental melon cultivars.

## 2 Materials and methods

### 2.1 Plant materials and growth conditions

A greenhouse experiment was conducted using two primary oriental melon cultivars (*Cucumis melo L*. var. makuwa Makino), which were seeded and cultivated during the winter of 2024 and spring of 2025. ‘Green Gem’ (A cultivar) and ‘Young White Lady’ (B cultivar). These cultivars were supported by Huzhou Wuxing Youtian Seed Industry Co., Ltd (Huzhou, China). The temperature in the seedling greenhouse was controlled at ≥24 °C during the day and ≥15 °C at night, with humidity maintained at approximately 65%. The trials were conducted in Jinnong Data-Driven Fruit and Vegetable Industry Base from Jinnong Ecol Agr Dev Co Ltd (30°82′N, 120°22′E), Huzhou, Zhejiang, China. As shown in **Table 1**, **Figure A1**, six LED light supplementation treatments were applied: (1) red: blue ratio 1:1, 18 W; (2) red: blue ratio 5:1, 18 W; (3) white light, 18 W; (4) control (CK, without light supplementation); (5) white light, 48 W; (6) white light, 60 W. The LED light was T8 type, and purchased from More Better Co.(Hangzhou, China). The lighting conditions were measured by Prometheus-P4 (Torch Bearer Co., Hangzhou, China). We collected the average lighting conditions parameters at 11:00-12:00. All light supplementary treatments were controlled at 12 hours. Four seedling seasons were replicated during 2024–2025, with seeding and sprouting dates as follows: December 20, 2024 (Season 1, S1); January 9, 2025 (Season 2, S2); January 19, 2025 (Season 3, S3); and February 11, 2025 (Season 4, S4). Oriental melon seeds were germinated in darkness for a period of three days. Seedlings were transferred to the LED supplemental lighting system once the sprouting rate exceeded 90%, and the light treatment was maintained for the following 30 days. Then, at 31 days after sowing, the seedlings were transplanted into the cultivation greenhouse. Pollination and pruning were performed at 110 days after sowing. The oriental melons were cultivated under protected cultivation with plastic greenhouse, rows were arranged at 1.0 m intervals and plants spaced 0.30 m within rows, yielding a designed density of D = 3.3 × 104 plants ha^-1^. The application of water-soluble fertilizers (20:20:20, Stanley Co., Linyi, China) was 45 kg·ha^-1^. Other plant protection and management practices were consistent with those used in local production. The oriental melons were harvested at 140–150 days after seeding. Each treatment was replicated with 20 plants during a single growing season.

**Table 1:** The LED light supplementary treatments during seedling stage.

Treatments	Power	Spectrum	Photoperiod
4(CK)	–	–	–
1	18 W	R:B= 1:1	16 hours
2	18 W	R:B= 5:1	16 hours
3	18 W	Full-spectrum	16 hours
5	48 W	Full-spectrum	16 hours
6	60 W	Full-spectrum	16 hours

### 2.2 Sensory evaluation

The sensory evaluation protocol was adapted from Bianchi et al. (2016). Sensory evaluation attributes and their definitions are listed in **Table 2**. A trained and analytical sensory panel, well-versed in the sensory assessment of agricultural products, evaluated the fruit from all treatments. The tasters were college students (aged 18–25 years) from Zhejiang A&F University. The panel comprised regular members of our sensory team, who underwent a week-long reorientation training (five sessions of 1 hour each) to focus on the sensory evaluation of oriental melons. This training utilized ISO 8586-2012 (ISO, 2012), and a selection of test samples to ensure consistency. Throughout the training, panelists received feedback on their scoring of attributes, which helped maintain the panel’s overall consistency. The trained panel consisted of 20 members. Five healthy melons were selected; each melon was longitudinally sectioned into 1-cm-thick slices along its central axis. The oriental melons samples were peeled, and halved melons were presented to panelists individually and identified using three-digit codes. Evaluations were conducted in individual sensory booths maintained at an ambient temperature of 20 °C, with daylight-corrected lighting employed to ensure optimal assessment conditions. The sensory data were standardized by benchmarking all scores against the control (CK). The control sample’s scores were anchored at 0 for all attributes. Each treatment sample was then rated on a relative scale from -10 to +10, where a positive value denotes a perceived intensity greater than the control, and a negative value denotes a lower perceived intensity. This approach controls for individual differences in scale usage and directly quantifies the treatment effect as a deviation from the control.

**Table 2:** Sensory evaluation attribute and their definitions. .

Attribute	Definition
Bitter	The bitter taste perceived through the taste organ.
Astringency	The astringent taste perceived through the taste organ.
Aroma	The odor of raw materials was assessed through the olfactory organ.
Sweetness	The sensation elicited by the stimulation of carbohydrate substances was perceived through the taste organ.
Texture	It describes the physical sensation a food or liquid creates in the mouth. It’s not about taste (sweet, sour) but about feel.
Appearance	Light perception obtained through visual organs, such as dark and light.
Overall acceptability	Subjective purchase intention regarding the sample.

### 2.3 Total soluble solids content

The determination of total soluble solids content (TSS) measured by the method of ISO 2173:2003 (ISO, 2003). The refractive index of a test solution is measured at 20 °C using a refractometer. The refractive index is correlated with the number of soluble solids, expressed as sucrose concentration, either by consulting tables or through direct reading on the instrument. The refractometer used should be capable of indicating either the refractive index or the mass fraction of sucrose. It is essential to calibrate the instrument such that it registers a refractive index of 1.333 for distilled water at 20 °C.

### 2.4 Soluble sugar content

The healthy oriental melons, weighing 350–550 g per melon for the Green Gem cultivar and 100–350 g for the Young White Lady cultivar, were chosen. Subsequently, each melon was longitudinally sectioned into slices that were 1 cm thick along its central axis, with this procedure being replicated three times. The slices were evenly divided using the quartering method and quickly soaked in liquid nitrogen for 10 minutes, then transferred to -80 °C for 72 hours. Soluble sugar content (SSC) was measured by the method of Grabowski et al. (2008), which employs anthrone colorimetry. A glucose standard solution was prepared and diluted to different concentrations. Anthrone reagent and concentrated sulfuric acid were then added, and the mixture was heated in a boiling water bath, cooled, and colorimetric measurements were taken at a wavelength of 620 nm to construct the standard curve. Melon samples were extracted using an 80% ethanol aqueous solution by low-temperature ultrasonic oscillation. The resulting extract was processed and colorimetric analysis was performed based on the standard curve. The absorbance was measured, and the soluble sugar content in the sample was determined using the standard curve.

### 2.5 Titratable acid content and sugar-acid ratio

The determination of titratable acid content (TA) was measured by the method of Shi et al. (2015). The juice was extracted from the edible pulp tissue of the melons. TA was measured using potentiometric titration with 0.1 N NaOH until a pH of 8.1 was reached. For this procedure, 1 ml of diluted juice was mixed with 25 ml of distilled water. The results were expressed as grams of citric acid equivalents per 100 g of fresh weight (FW). For the analysis of these parameters, three melons from each treatment group were utilized. The sugar-acid ratio was calculated by dividing the soluble sugar content by the titratable acid content.


Sugar−acid ratio= Soluble sugar content / Titratable acid content

### 2.6 Vitamin C content

The determination of Vitamin C content (Vc) was measured by the method of Silveira et al. (2013). Frozen sample (10 g) was homogenized with 10 mL extraction solution (19.2 g·L^-^¹ citric acid, 0.5 g·L^-^¹ EDTA-Na_2_, 50 mL·L^-^¹ methanol, 1.68 g·L^-^¹ NaF) for 30 s and filtered. The filtrate was adjusted to pH 2.40 and centrifuged at 10, 500× g for 5 min at 5 °C. Supernatant was passed through a methanol-activated C18 cartridge and 0.45 μm membrane. For dehydroascorbic acid (DHAA) derivatization, 1 mL of 1, 2-phenylenediamine (35 mg·100 mL^-^¹) was added to 3 mL extract and kept in dark for 37 min. A 20 μL aliquot was injected into HPLC equipped with C18 column (30 cm × 3.9 mm, 5 μm). Mobile phase was methanol: water (5:95, v/v) containing 1.82 g·L^-^¹ hexadecyltrimethylammonium bromide and 6.8 g·L^-^¹ KH_2_PO_4_ (pH 4.59) at 1.0 mL·min^-^¹, 25 °C. Detection wavelengths were 245 nm for ascorbic acid (AA) and 348 nm for DHAA. Vitamin C content was calculated as AA + DHAA and expressed as mg·100 g^-^¹ fresh weight.

### 2.7 Moisture content

The moisture content was calculated according to Yu et al. (2023). Three healthy melons were selected. The melons were washed thoroughly, air-dried for 30 minutes, and sliced into 0.5 cm thick pieces using a precision slicer. The slices were divided into four equal portions using the quartering method, and three of these portions were randomly selected as replicates. The samples were weighed and dried in a forced-air oven at 80 °C until reaching a constant weight. Moisture content was determined using the specified formula:


$$\text{Moisture content }(\%)=(\frac{\left(\text{Weight before drying}-\text{Weight after drying}\right)}{\text{Weight before drying}})\times 100\%$$

### 2.8 Textural properties

Three healthy oriental melons (350–550 g per melon for Green Gem, 100–350 g for Young White Lady) were selected, each melon was longitudinally sectioned into slices 1 cm in thickness along its central axis. The textural properties of the melon’s flesh were assessed using a texture profile analysis (TPA) protocol that was adapted from the methods of Bianchi et al. (2016); Xu et al. (2023). The measurements were conducted with a physical property analyzer (model TMS-PRO, FTC company). The textural properties of slices were measured using a P/5 probe at the center of each slice. The resulting curve provided parameters such as firmness, adhesion, cohesion, springiness, and chewiness. The parameters were set as follows: the pretest speed was 30 mm/min, the test speed was 60 mm/min, and the posttest speed was 90 mm/min. The compression ratio was set at 60%, with a 5 second pause between tests, and the trigger force was 0.2 N. Each sample was measured in five replicates. The highest and lowest values from each replicate were discarded, and the average value was calculated.

### 2.9 Statistical analysis

Statistical analyses were conducted in SPSS 23.0 (IBM, USA), employing one-way ANOVA with Tukey’s honestly significant difference (HSD) test, Spearman rank correlation analysis and Principal Component Analysis (PCA). Table data were generated using Microsoft Excel 2018 (Microsoft Corp., USA), while graphical representations were plotted in Origin 2025 Professional (OriginLab Corp., USA).

## 3 Results

### 3.1 Sensory attributes

**Figure 1:**
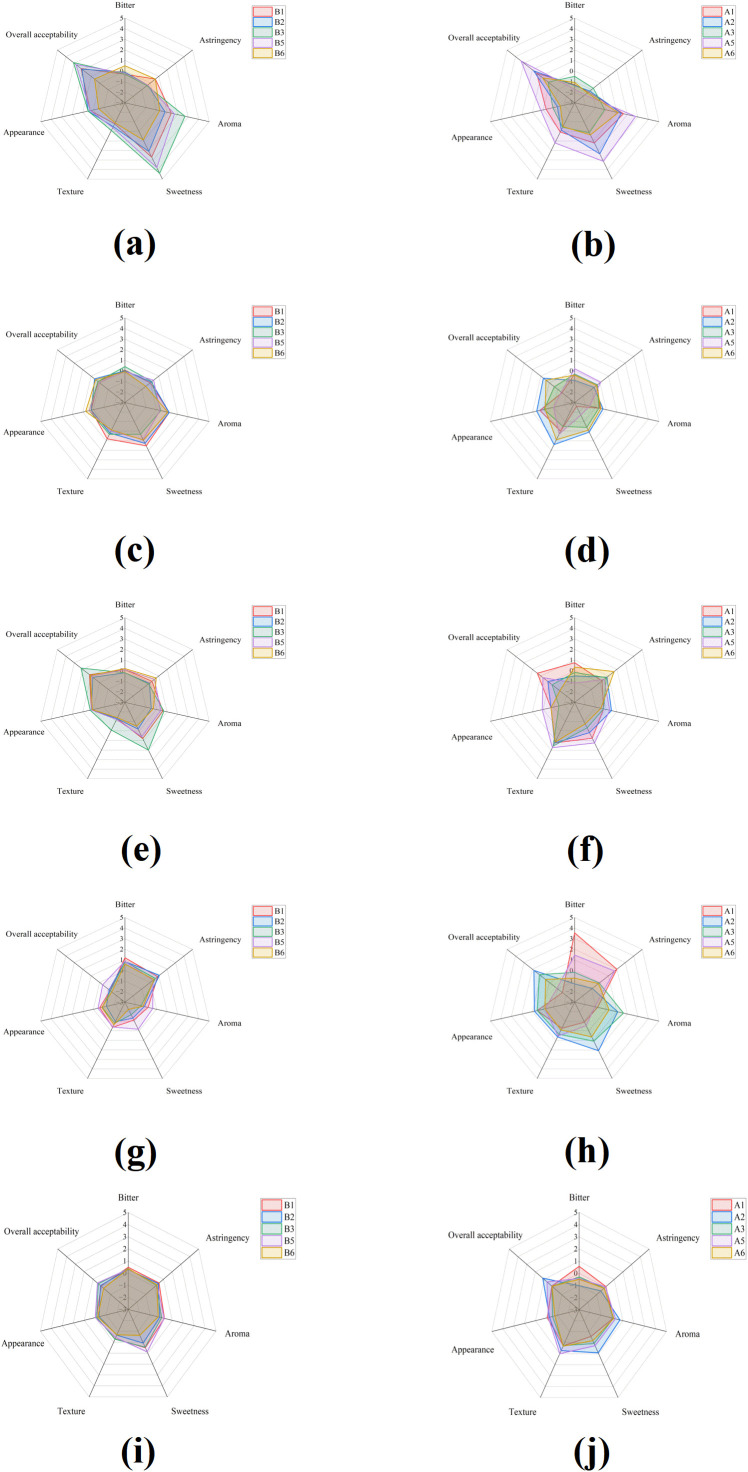
The effect of LED Supplementary Lighting on sensory profile of oriental melon fruit. **(a)** Green Gem in total seasons; **(b)** Young White Lady in total seasons; **(c)** Green Gem in season 1; **(d)** Young White Lady in season 1; **(e)** Green Gem in season 2; **(f)** Young White Lady in season 2; **(g)** Green Gem in season 3; **(h)** Young White Lady in season 3; **(i)** Green Gem in season 4; **(j)** Young White Lady in season 4. The sensory evaluation values of the control groups (A4 and B4) were regarded as 0 score for all profile.

The sensory attributes of two melon cultivars were evaluated under various LED supplementary lighting treatments during the seedling stage (**Figure 1**). The sensory evaluation values of the control groups (A4 and B4) were regarded as 0. The sensory parameters assessed included overall acceptability, bitterness, appearance, texture, sweetness, aroma, and astringency, using a relative scoring system based on comparison to the CK. In ‘Green Gem’, no significant differences were found across treatments in overall acceptability, appearance, texture, aroma, or astringency (*p* > 0.05) (**Figure 1a**). This can be ascribed to the considerable sensory variation resulting from individual differences in oriental melons.

Meanwhile, the sweetness score of A2 (1.26 ± 2.45) was significantly higher than that of A1 (-0.21 ± 2.54, *p* < 0.05). Across all seasons, no significant differences were observed among treatments for overall acceptability, bitterness, appearance, texture, aroma, or astringency (*p* > 0.05) in ‘Young White Lady’ (**Figure 1b**). In contrast, the sweetness score of B5 (0.88 ± 2.11) was higher than that of B6 (0.52 ± 2.44, *p <* 0.05).

By combining data from all four seasons, it was evident that the standard deviations were high, indicating pronounced seasonal variation (**Figures 1c–j**). The bitterness scores ranked as S1 (0.56) > S2 (-0.15) > S3 (-0.34) > S4 (-1.19), with S1 significantly more bitter than S4 (*p <* 0.05). For astringency, the scores followed the order S1 (0.80) > S2 (0.51) > S3 (-0.32) > S4 (-1.33), and S1 was significantly higher than both S3 and S4 (*p <* 0.05). Aroma and sweetness were highest in S4 (1.37 and 1.44, respectively), significantly exceeding S1, S2, and S3 (*p <* 0.05). Texture was significantly higher in S2 (1.40) than in other seasons, while appearance was greatest in S1 (0.31). Overall acceptability score was highest in S4 (1.53), surpassing the other seasons (*p <* 0.05). The more optimal growth conditions in S3 & S4, characterized by a combination of suitable light intensity, were particularly beneficial for oriental melons growing (**Supplementary Figures S1**, **S2**).

### 3.2 Impact of LED supplementary lighting during the seedling stage on total soluble solids and soluble sugar content

**Figure 2:**
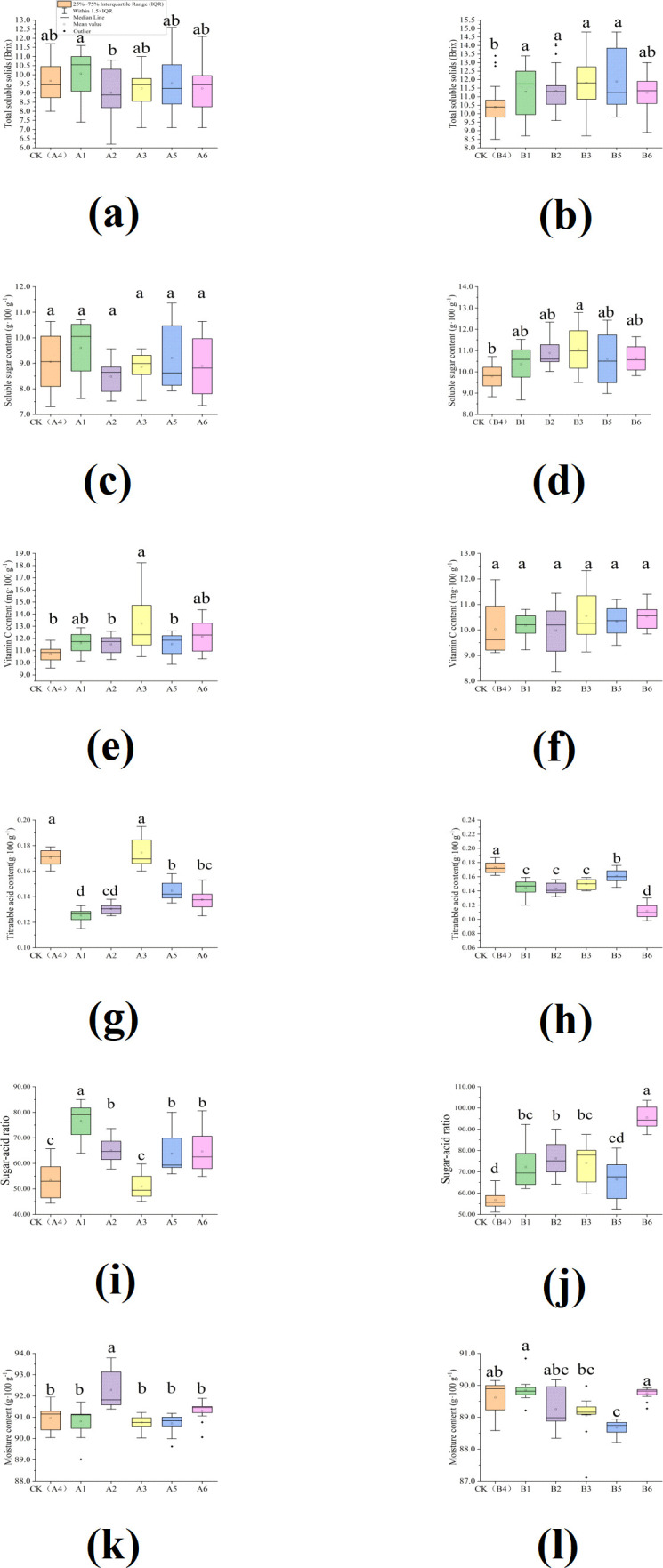
Effects of LED light supplementary treatments during seedling stage on total soluble solids(TSS), soluble sugar content(SSC), Vitamin C(VC), Titratable acid content(TAC), Sugar-acid ratio, and Moisture content in total seasons: **(a)** TSS of Green Gem; **(b)** TSS of Young White Lady; **(c)** SSC of Green Gem;**(d)** SSC of Young White Lady; **(e)** Vc of Green Gem; **(f)** Vc of Young White Lady;**(g)** TAC of Green Gem; **(h)** TAC of Young White Lady;**(i)** sugar-acid ratio of Green Gem; **(j)** sugar-acid ratio of Young White Lady;**(k)** moisture content of Green Gem; **(l)** moisture content of Young White Lady. Different lowercase letters in the figure indicate significant differences (*p <* 0.05) for the LED treatments in this cultivar, as determined by Tukey’s honestly significant difference test.

Sweetness is a key driver of melon quality and consumer preference, and is primarily measured as total soluble solids (TSS) and soluble sugar content (SSC) (Tzuri et al., 2025). As **Figures 2a, b** shown, the response of TSS to LED supplementary lighting was cultivar dependent. In ‘Green Gem’, the overall TSS content was not markedly different from CK (9.7 ± 1.1°Brix), though a significant difference was observed between specific treatments, with A1 (10.1 ± 2.0°Brix) being statistically superior to A2 (9.0 ± 1.1°Brix). In contrast, for ‘Young White Lady’, a consistent and significant enhancement in TSS (*p <* 0.05) was observed under all LED treatments throughout the growing seasons (**Figure 2b**). The most notable increase was seen in the B5 treatment (11.9 ± 1.5°Brix), which was significantly greater than that of CK (10.4 ± 1.0°Brix, *p <* 0.05). This may be due to increased soluble solids or reduced moisture content. The considerable variation, as indicated by the high standard errors, can be attributed to seasonal differences across the study periods.

The impact of LED supplementary lighting during the seedling stage on the soluble sugar content of the two melon cultivars was analyzed: ‘Green Gem’ and ‘Young White Lady’ (**Figures 2c, d**). The SSC for ‘Green Gem’ across all seasons appears to be slightly influenced by the LED supplementary lighting treatments (*p >*0.05). For ‘Young White Lady’, SSC was either slightly enhanced or stable under LED treatments; only the B3 treatment (11.06 ± 1.58 g·100 g^-1^) showed a significant increase compared to CK (9.79 ± 0.81 g·100 g^-1^, *p* < 0.05), while other treatments did not differ significantly from CK (*p* > 0.05).

### 3.3 Impact of LED supplementary lighting during the seedling stage on vitamin C and titratable acid content

The effects of LED lighting during the seedling stage on the vitamin C content (Vc) of two melon cultivars, ‘Green Gem’ and ‘Young White Lady’, were investigated (**Figures 2e, f**). For ‘Green Gem’, the A3 treatment (13.23 ± 4.25 mg·100 g^-1^) exhibited a significant increase in Vc content compared to the CK (10.70 ± 0.77 mg·100 g^-1^) across all growing seasons (*p <* 0.05). No other LED treatments showed significant differences from the CK (*p >* 0.05). In contrast, for ‘Young White Lady’, none of the LED treatments resulted in a significant difference in Vc content compared to the CK in any season (*p >* 0.05), indicating a weaker response of this cultivar to supplemental lighting during the seedling stage. Notably, this result differs from previous reports on kiwifruit, where a mixed red and blue light treatment was found to be effective in enhancing Vc concentration, suggesting a species specific or stage response (Wang et al., 2022).

Supplemental LED lighting during the seedling stage consistently suppressed titratable acid accumulation in both melon cultivars (**Figures 2g, h**). Across all seasons and treatments, with the sole exception of A3 in ‘Green Gem’, every LED regimen significantly reduced titratable acid content compared to the non-LED control (CK) (*p <* 0.05). The uniformity and statistical strength of this reduction across two genetically distinct cultivars strongly suggest that seedling-stage LED supplementation exerts a robust inhibitory effect on acid buildup.

### 3.4 Impact of LED supplementary lighting during the seedling stage on sugar-acid ratio and moisture content

The sugar-acid ratio, determined by the soluble sugar content and titratable acidity, is widely used to evaluate melon quality (Zhang et al., 2025). Supplemental LED lighting during the seedling stage significantly boosted the sugar-acid ratio in two cultivars (**Figures 2i, j**). Compared to the CK (A4, 53.3 ± 9.2), all LED treatments except A3 significantly in-creased the sugar-acid ratio in ‘Green Gem’ (*p <* 0.05), with A1 (76.6 ± 9.8) showing the highest value. Similarly, all LED treatments significantly enhanced the ratio in ‘Young White Lady’ over CK (B4, 56.8 ± 6.9), with B6 (95.5 ± 7.0) being the most effective. This enhancement was primarily due to a reduction in titratable acidity.

**Figure 3:**
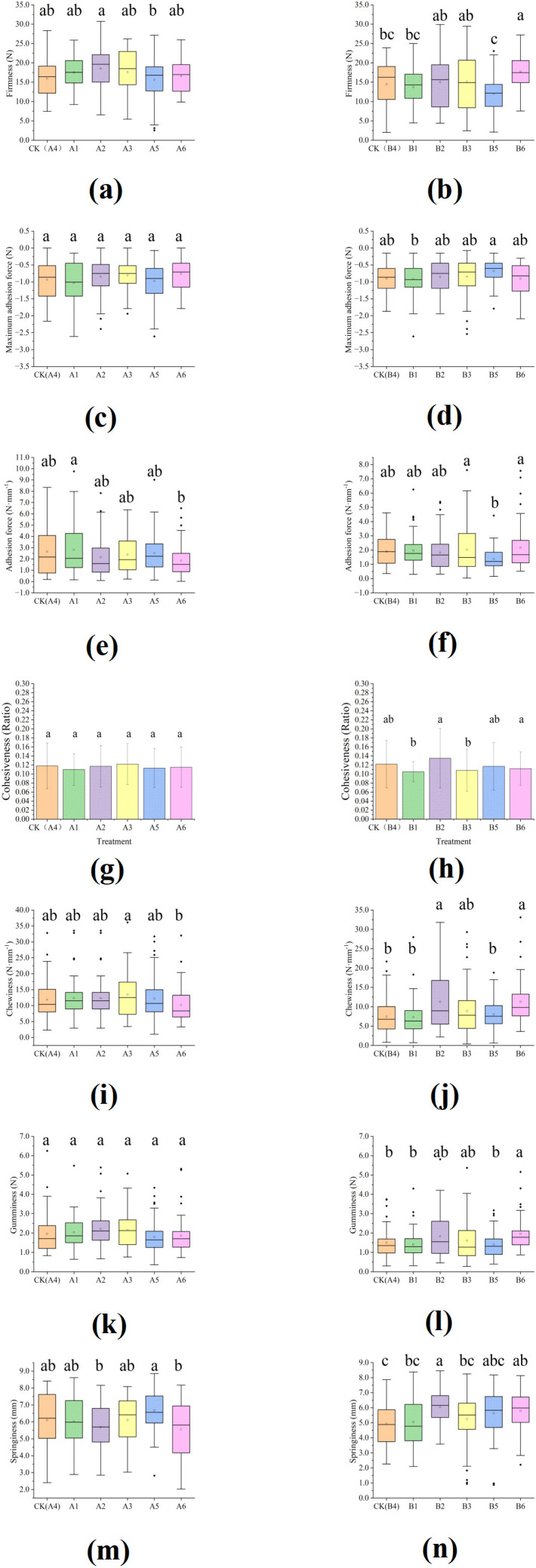
Effects of LED light supplementary treatments during seedling stage on Textural properties: **(a)** Firmness of ‘Green Gem’ in total seasons; **(b)** Firmness of ‘Young White Lady’ in total seasons; **(c)** Maximum adhesion force of ‘Green Gem’ in total seasons; **(d)** Maximum adhesion force of ‘Young White Lady’ in total seasons; **(e)** Adhesion force of ‘Green Gem’ in total seasons; **(f)**Adhesion force of ‘Young White Lady’ in total seasons; **(g)** Cohesiveness of ‘Green Gem’ in total seasons; **(h)** Cohesiveness of ‘Young White Lady’ in total seasons; **(i)** Chewiness of ‘Green Gem’ in total seasons; **(j)** Chewiness of ‘Young White Lady’ in total seasons; **(k)** Gumminess of ‘Green Gem’ in total seasons; **(l)** Gumminess of ‘Young White Lady’ in total seasons; **(m)** Springiness of ‘Green Gem’ in total seasons; **(n)** Springiness of ‘Young White Lady’ in total seasons. Different lowercase letters in the figure indicate significant differences (*p <* 0.05) for the LED treatments in this cultivar, as determined by Tukey’s honestly significant difference test.

Moisture content was an important factor for melon quality, and demonstrated a strong correlation with flesh firmness (Yang et al., 2025). The influence of LED supplemental lighting during the seedling stage on the moisture content of fruits of two melon cultivars is summarized in **Figures 2k, l**. In ‘Green Gem’, the A2 treatment consistently showed a significant increase in moisture content (92.29 ± 1.37 g·100 g^-1^) over the CK (90.84 ± 0.68 g·100 g^-1^) in all seasons (*p <* 0.05), indicating a specific positive effect of this light regimen. The remaining LED treatments did not differ significantly from the CK (**Figure 3a**). In ‘Young White Lady’, a significant reduction in moisture content was specific to the B5 treatment (88.68 ± 0.38 g·100 g^-1^ vs. CK: 89.62 ± 8.29 g·100 g^-1^; *p <* 0.05), which may reflect decreased intercellular hydration. Non-significant variations in other treatments (e.g., B2, B3) are attributable to inherent biological variability among individual plants.

### 3.5 Effects of LED light supplementary treatments during seedling stage on textural properties

Texture is a critical quality attribute in the evaluation of oriental melons (Pan et al., 2022). Consumer preference for fruit pulp is defined by a balanced firmness (Lázaro and de Lorenzo, 2015). We analyzed the effect of LED supplemental lighting during the seedling stage on the firmness of the sweet melon cultivars ‘Green gem’ and ‘Young White Lady’ at harvest (**Figure 3**). In ‘Green Gem’, the firmness of A2 (18.55 ± 4.63 N) was significantly higher than A5 (15.63 ± 5.96 N, *p <* 0.05), and no LED treatment differed significantly from the CK (A4). The remaining LED treatments did not differ significantly from CK. For ‘Young White Lady’, no significant differences from CK(B4, 14.50 ± 8.29 N) were observed in LED treatments except B6 (**Figure 3b**). The firmness of B6 (17.74 ± 3.45 N) conferred significantly superior firmness relative to CK and B5 (12.00 ± 5.59 N, *p <* 0.05). This enhanced firmness in B6 treatment suggests greater structural integrity and density of the fruit tissue, as well as stronger intercellular bonding, commonly resulting from a lower degree of fruit maturity. As shown in **Figures 3c–f**, the maximum adhesion force and the adhesion force under LED lighting treatments were not significantly different from those under CK treatments (*p >* 0.05) in both cultivars. The cohesiveness of the two melon cultivars under seedling-stage LED lighting is presented in **Figures 3g, h**. No significant differences were observed between any LED treatment and their respective controls (CK A4 for Green Gem; CK B4 for ‘Young White Lady’) across all growing seasons (*p >* 0.05). However, for ‘Young White Lady’, significant differences were detected among some LED treatments: the cohesiveness values of B1 and B3 were significantly lower than that of B2 when data from all seasons were pooled. The effects of supplemental LED lighting during the seedling stage on the chewiness of melon fruits are shown in **Figures 3i, j**. For the cultivar ‘Green Gem’, chewiness under treatments A3 (1.85 ± 0.42 N·mm^-1^) and A6 (2.14 ± 0.59 N·mm^-1^) differed significantly from each other (*p <* 0.05), but neither was significantly different from the CK (A4: 1.81 ± 0.62 N·mm^-1^) across all seasons. All other LED treatments also showed no statistically significant difference in chewiness compared to the CK (*p >* 0.05). For ‘Young White Lady’, a significant increase in chewiness was observed in treatments B2 (1.84 ± 0.94 N·mm^-1^) and B6 (1.96 ± 0.75 N·mm^-1^) relative to the CK (1.49 ± 0.67 N·mm^-1^; *p <* 0.05). The remaining LED treatments did not result in any significant difference in chewiness compared to the control in total seasons. The gumminess of two melon cultivars as **Figures 3k, l** shown. There were no significant differences in gumminess were observed between any LED treatment and the CK(A4) of ‘Green Gem’ in total seasons (*p >* 0.05). In ‘Young White Lady’, the gumminess of B6 (1.96 ± 0.75 N) was significantly higher than CK (B4, 1.49 ± 0.67 N, *p <* 0.05) in total seasons. The springiness of two melon cultivars as **Figures 3m, n** shown. In total seasons, no LED treatments significantly affected the springiness of ‘Green Gem’ compared to CK (*p >* 0.05). In contrast, for ‘Young White Lady’, the springiness of B2 (6.05 ± 0.67 mm) was significantly higher than that of B6 (5.80 ± 1.40 mm). However, neither B2 nor B6 differed significantly from the CK (B4, 4.94 ± 0.80 mm, *p <* 0.05). This suggests that ‘Young White Lady’ is more responsive to LED lighting than ‘Green Gem’.

### 3.6 Correlation analysis

**Figure 4:**
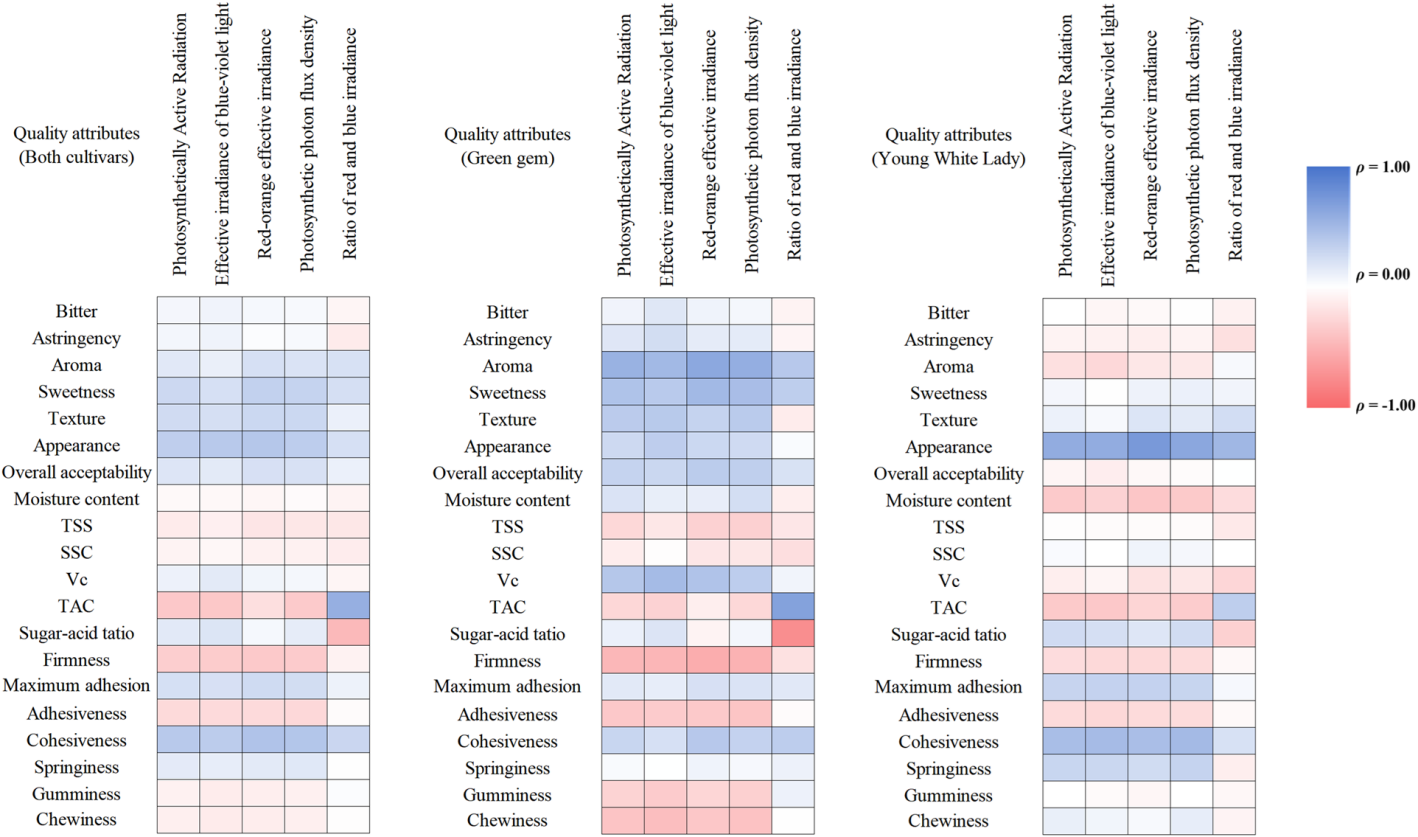
Spearman rank correlation analysis between light parameters with melon quality attributes.

Spearman rank correlation analysis was employed to assess monotonic relationships between light parameters and melon quality attributes as **Figure 4** shown. Cohesiveness exhibited the strongest monotonic relationship with red-orange effective irradiance (ρ = 0.435, *p <* 0.01) and was also strongly correlated with photosynthetic photon flux density (PPFD) (ρ = 0.417, *p <* 0.01) and photosynthetically active radiation (PAR) (ρ = 0.390, *p <* 0.05). Appearance score was strongly correlated with both red-orange effective irradiance (ρ = 0.409, *p <* 0.01) and blue-violet effective irradiance (ρ = 0.390, *p <* 0.01). These results indicate that higher ranks of both red-orange and blue-violet light intensities at seedling stage are associated with superior ranks in fruit cohesiveness and visual appearance at harvest day. Several other quality attributes demonstrated significant, though slightly weaker, positive correlations. Sweetness was positively correlated with red-orange effective irradiance (ρ = 0.334, *p <* 0.05) and PPFD (ρ = 0.321, *p <* 0.05). Texture score showed similar correlations with red-orange effective irradiance and PPFD (ρ = 0.287, *p <* 0.05). The ratio of red-to-blue irradiance (R:B ratio) showed a pronounced and inverse relationship with key chemical traits, while fruit firmness was negatively correlated with broad-spectrum light intensity. TAC increased monotonically with a higher R:B ratio (ρ = 0.579, *p <* 0.01). Conversely, the Sugar-Acid Ratio decreased significantly as the R:B ratio increased (ρ = -0.465, *p <* 0.01). Firmness was significantly negatively correlated with multiple light parameters, including red-orange effective irradiance (ρ = -0.357, *p <* 0.05), PPFD (ρ = -0.342, *p <* 0.05), and blue-violet effective irradiance (ρ = -0.338, *p <* 0.05).

Separate correlation analyses were conducted for each cultivar to prevent potential confounding by Simpson’s paradox. In ‘Green Gem’, fruit quality was co-influenced by light intensity and spectral composition, albeit with contrasting effects. High-intensity broad-spectrum lighting—quantified as Photosynthetically Active Radiation (PAR) and Photosynthetic Photon Flux Density (PPFD)—was significantly and positively correlated with aroma, sweetness, and Vc content, but significantly and negatively correlated with fruit firmness, revealing an intrinsic trade-off. Notably, the Red-to-Blue irradiance ratio (R:B Ratio) independently and dominantly governed flavor formation: a high R:B ratio sharply increased TAC while substantially decreasing the sugar-acid ratio, thereby exerting a strong negative impact on flavor. In contrast, ‘Young White Lady’ exhibited a more targeted response pattern. Its quality improvement was primarily driven by high-intensity broad-spectrum light, particularly red-orange irradiance, which strongly correlated with enhanced appearance and cohesiveness. However, flavor attributes (e.g., aroma and sweetness) and most physicochemical properties showed minimal correlation with light parameters. The influence of the R:B Ratio was also limited and non-significant, indicating that its flavor profile remains relatively insensitive to spectral variation.

The results demonstrated that LED supplemental lighting during the seedling stage may exert a significant and lasting influence on mature fruit quality. The enhancement of sensory attributes (appearance, sweetness) and textural properties (cohesiveness) is primarily driven by increased blue-violet and red-orange irradiance. Conversely, a high red-to-blue ratio promotes acid accumulation and reduces firmness. For ‘Green Gem’, lighting strategies must balance flavor enhancement against textural optimization, whereas for ‘Young White Lady’, management can focus on leveraging light intensity to maximize visual commodity traits. These findings confirm that targeted spectral management at the seedling stage is a viable strategy for precisely regulating the nutritional and sensory profile of melons at harvest.

### 3.7 PCA analysis

The principal component analysis was performed on eating quality and textural properties index based on the initial data, extracting 6 principal components from the feature values. The contribution rates of these components were 25.767%, 18.294%, 12.049%, 10.288%, 8.200% and 6.541%, with a cumulative contribution rate of 81.139% (**Table 3**). Only PCs 1–6 were retained as their cumulative variance exceeds 81%, while components 7–14 were excluded due to their individual variance being less than 5%.

**Table 3:** Principal component analysis of variance.

Components	Initial eigenvalue			Extracting square sum and loading		
	Total	Variance/%	Accumulate/%	Total	Variance/%	Accumulate/%
PC1	5.153	25.767	25.767	5.153	25.767	25.767
PC2	3.659	18.294	44.061	3.659	18.294	44.061
PC3	2.410	12.049	56.111	2.410	12.049	56.111
PC4	2.058	10.288	66.398	2.058	10.288	66.398
PC5	1.640	8.200	74.599	1.640	8.200	74.599
PC6	1.308	6.541	81.139	1.308	6.541	81.139
PC7	0.922	4.610	85.749			
PC8	0.808	4.042	89.791			
PC9	0.610	3.049	92.840			
PC10	0.455	2.274	95.114			
PC11	0.292	1.462	96.576			
PC12	0.256	1.281	97.858			
PC13	0.148	0.739	98.596			
PC14	0.084	0.422	99.019			
PC15	0.074	0.370	99.388			
PC16	0.064	0.318	99.706			
PC17	0.024	0.122	99.828			
PC18	0.019	0.094	99.922			
PC19	0.011	0.055	99.977			
PC20	0.005	0.023	100.000			

As shown in **Table 4**, PC1 which accounted for 25.77% of the variance, emerged as the primary dimension governing quality differentiation. It was characterized by a fundamental trade-off between textural properties and sensory attributes. Textural properties, including firmness, adhesiveness, and chewiness (loadings > 0.3), were strongly and inversely associated with sensory attributes such as overall acceptability, sweetness, and aroma (loadings < -0.3), revealing an inherent conflict between structural integrity and flavor desirability. PC2 accounted for 18.29% of the variance, it constituted a sugar accumulation dimension, predominantly defined by TSS, SSC, and the sugar-acid ratio (loadings > 0.3). The third component PC3 was 12.05% variance, and it represented a synergistic dimension of flavor and texture, with joint contributions from aroma, sweetness, and texture. Subsequent components delineated independent dimensions related to undesirable flavors and appearance (PC4), sugar-acid balance (PC5), and springiness texture (PC6).Principal component coefficients (eigenvectors) were calculated by scaling the factor loadings of the 20 variables by the reciprocal of their eigen-values’ square roots (**Table 4**).

**Table 4:** The load values of principal components.

Index	PC1(Y_1_)	PC2(Y_2_)	PC3(Y_3_)	PC4(Y_4_)	PC5(Y_5_)	PC6(Y_6_)
Overall acceptability(X_1_)	-0.333	-0.050	0.339	-0.042	0.052	-0.010
Firmness(X_2_)	0.327	0.100	0.248	-0.180	-0.050	-0.295
Adhesiveness(X_3_)	0.322	0.048	0.257	0.060	-0.013	0.356
Sweetness(X_4_)	-0.321	-0.039	0.370	-0.038	0.034	-0.016
Chewiness(X_5_)	0.309	-0.151	0.216	-0.223	0.130	0.201
Gumminess(X_6_)	0.307	-0.048	0.346	-0.211	-0.005	-0.125
Texture(X_7_)	-0.269	0.031	0.299	0.256	0.109	0.055
Maximum adhesion force(X_8_)	-0.268	-0.146	-0.312	-0.032	0.067	-0.305
Aroma(X_9_)	-0.258	-0.049	0.429	0.101	0.012	-0.182
Bitter(X_10_)	0.229	0.108	0.079	0.483	0.011	-0.010
TSS(X_11_)	-0.106	0.460	0.002	-0.011	-0.067	0.115
SSC(X_12_)	-0.105	0.457	-0.004	0.000	0.034	0.156
Sugar-acid ratio(X_13_)	-0.024	0.381	-0.018	-0.068	0.515	-0.012
Moisture content(X_14_)	0.194	-0.308	0.030	0.093	0.255	-0.327
Cohesiveness(X_15_)	-0.152	-0.254	0.029	-0.129	0.059	0.243
Astringency(X_16_)	0.164	0.251	0.032	0.408	-0.071	-0.063
Appearance(X_17_)	-0.042	-0.237	0.151	0.425	0.138	0.158
Springiness(X_18_)	0.011	-0.226	-0.162	0.028	0.301	0.558
Vc (X_19_)	0.091	-0.123	-0.155	0.412	0.084	-0.165
TAC(X_20_)	-0.046	-0.107	0.021	0.090	-0.706	0.173

The principal components were then formulated as linear combinations using these coefficients:


PC_Total (Y_Total)=0.318×Y1＋0.225×Y2＋0.148×Y3＋0.127×Y4＋0.101×Y5＋0.081 ×Y6 

**Figure 5:**
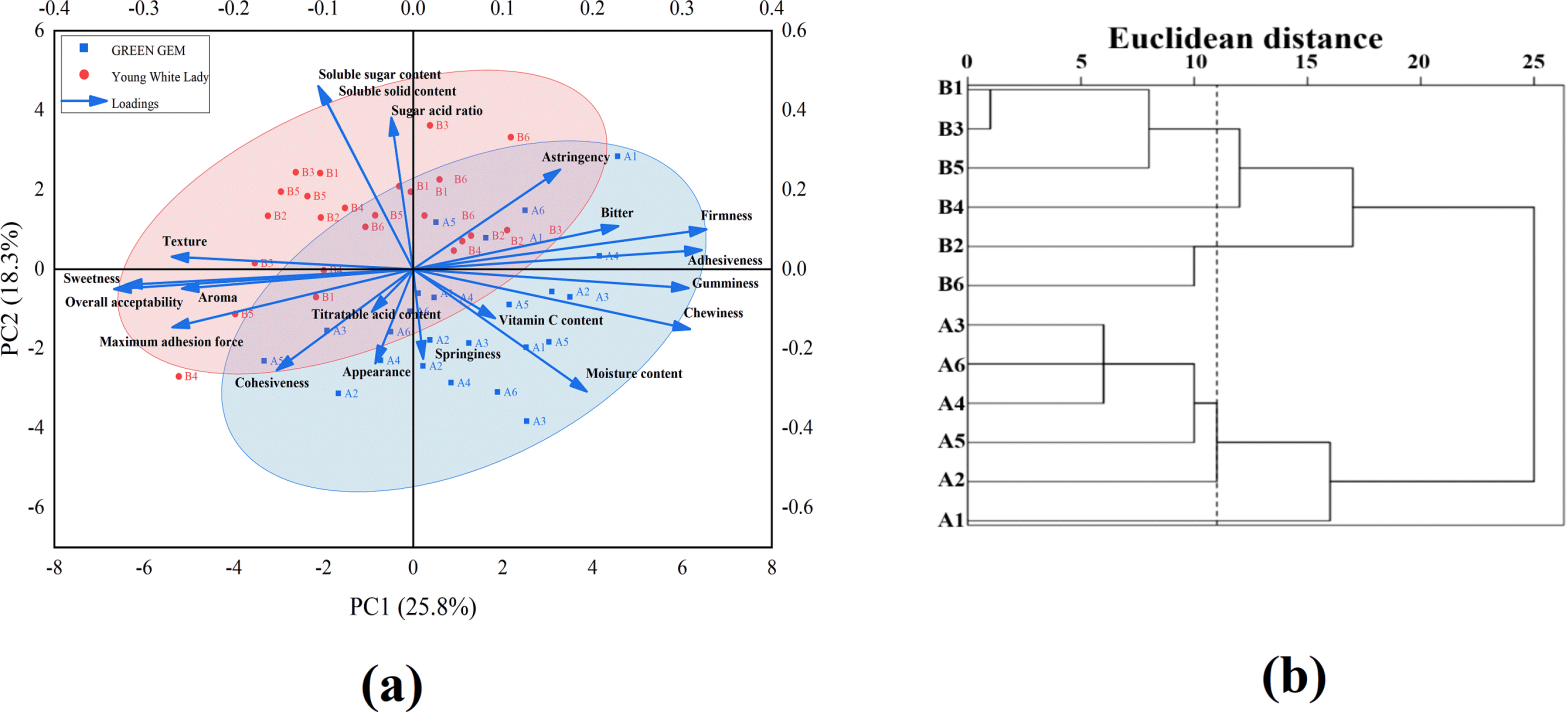
PCA biplot of sensory and physicochemical attributes and hierarchical cluster analysis. **(a)** PCA biplot. Vectors show variable loadings; points represent samples. PC1 and PC2 explain 25.76% and 18.29% of total variance, respectively; **(b)** Hierarchical cluster analysis. The clustering was performed using Z-score standardized data from 20 fruit quality attributes, with Euclidean distance and Ward’s method. All treatments were clearly segregated into two primary clusters, corresponding to the cultivars ‘Green Gem’ (A-series) and ‘Young White Lady’ (B-series), respectively.

A principal component biplot was constructed to visualize the distribution and correlation of fruit quality traits for the two melon cultivars in the reduced dimensional space (**Figure 5a**). The samples of ‘Green Gem’ were predominantly clustered in the positive region of PC1 and the negative region of PC2, closely associated with textural properties (hardness, adhesiveness) and undesirable flavors (bitterness, astringency). This distribution indicates that ‘Green Gem’ is characterized by a firm texture and pronounced bitter/astringent notes. In contrast, ‘Young White Lady’ samples were primarily located in the positive region of PC2 and the negative region of PC1, near vectors for TSS, soluble sugars, and the sugar-acid ratio, highlighting its superior sweetness and soluble solid content. The minimal overlap between the two cultivars in the PC1-PC2 plane demonstrates a significant divergence in their core quality profiles.

PC1 revealed a fundamental trade-off: textural properties (firmness, adhesiveness) were inversely associated with desirable sensory attributes (sweetness, overall acceptability). This suggests a potential dilemma in breeding and cultivation: selecting for superior texture might come at the cost of flavor intensity, and vice versa. PC2 was clearly a sugar accumulation dimension, strongly associated with TSS and SSC. The clustering of ‘Young White Lady’ samples in the positive PC2 region (**Figure 5a**) confirmed its genetic predisposition for high sugar content, which can be further amplified by appropriate lighting.

### 3.8 Hierarchical cluster analysis

Hierarchical cluster analysis clearly differentiated melon cultivars and revealed LED treatment effects, with genetic background being the primary factor (**Figure 5b**). Within each cultivar, sub-clusters indicated that specific LED spectra modulated fruit quality. For instance, A2 in ‘Green Gem’ was associated with superior sensory profiles, while B5 and B6 in ‘Young White Lady’ excelled in sweetness and textural properties, respectively. The clear divergence in response between ‘Green Gem’ and ‘Young White Lady’ with the former being more sensitive to spectral composition and the latter to light intensity highlights the importance of tailoring light recipes to genetic background. This aligns with the hierarchical clustering results, which clearly separated the two cultivars regardless of light treatment, underscoring genetics as the primary driver of quality variation (Tzuri et al., 2025).

## 4 Discussion

Seasonal low-light intensity and short photoperiod often lead to reduced yield, smaller fruit size, and inferior quality in horticultural crops, particularly during winter and spring (Paponov et al., 2019; Appolloni et al., 2021). To mitigate these challenges, many studies in facility agriculture have focused on increasing light intensity during reproductive growth. However, the present study provides evidence that LED supplemental lighting applied during the seedling stage exerts profound and cultivar-specific carry-over effects on the sensory, nutritional, and textural quality of oriental melon fruits at harvest. The seedling stage represents a critical window of physiological plasticity, during which light signals shape long-term plant development and final fruit quality by regulating photosynthetic apparatus development, carbon–nitrogen partitioning, and phytohormone balance (Zeng et al., 2025; Han et al., 2023). These findings extend our understanding of photomorphogenic regulation beyond immediate growth responses, demonstrating that the early light environment can shape developmental trajectories that influence fruit quality months later.

### 4.1 Spectral regulation of flavor-related metabolites: bitterness and sweetness

The significantly lower bitterness score in A2 (R:B = 5:1, 18 W) compared to A1 (R:B = 1:1, 18 W) (**Figure 1a**) points to a spectral-specific regulation of bitter compound biosynthesis. While both treatments shared the same light intensity, we hypothesize that the higher proportion of red light in A2 likely suppressed the accumulation of cucurbitacin, notably cucurbitacin B (Luo et al., 2024). This finding aligns with the established role of blue light and UV radiation in activating defense-related secondary metabolites through specific signaling cascades, including the ROS-Ca^2+^-WRKY pathway (Zhang et al., 2021; Jadidi et al., 2023). Our results suggested that reducing the blue light fraction during seedling development may suppress the transcriptional activation of cucurbitacin biosynthetic genes, a mechanism well-documented in related cucurbits. Moreover, the spectral-specific regulation observed in our study may involve photoreceptor-mediated control of transcription factors. Red LED light supplementation at 30 days after anthesis induced *CmHY5* and *CmWRKY28* to activate the expression of target genes, thereby promoting sucrose accumulation in melon fruit (Guan et al., 2026). Red LED light supplementation suppressed *CmPIF8* expression, which relieved its inhibition of *CmACO1* and enhanced both 1-aminocyclopropane-1-carboxylate oxidase and sucrose phosphate synthase activities, thereby promoting ethylene production and sucrose accumulation (Guan et al., 2024). Additionally, red light regulated ester biosynthesis through the *CmPIF8-CmMYB113/CmDof13* molecular module, enhancing the aroma of fruit (Wang et al., 2026). The cultivar-dependent response in soluble solids accumulation—with ‘Young White Lady’ showing consistent TSS enhancement across all LED treatments while ‘Green Gem’ exhibited minimal response—highlights the genetic basis of light responsiveness. This differential responsiveness may reflect variation in the expression or sensitivity of photoreceptors and downstream signaling components between cultivars (Ma et al., 2025). The high standard deviations observed across seasons in our TSS and SSC measurements reflect the complex interaction between genetic predisposition, light treatment, and environmental conditions, consistent with the understanding that fruit quality attributes are governed by multivariate interactions (Vallone et al., 2013).

### 4.2 Textural properties modulation and cell wall dynamics

The significant enhancement of firmness, chewiness, and gumminess under specific light regimens, particularly A2 in ‘Green Gem’ and B6 in ‘Young White Lady’ suggested that seedling-stage light spectrum programed cell wall architecture and metabolism in developing fruits. Textural properties are fundamentally determined by cell wall composition, including pectic substances, cellulose, hemicellulose, and structural proteins, as well as by turgor pressure. The negative correlation between broad-spectrum light intensity and firmness observed in our correlation analysis suggests a potential trade-off between photosynthetic carbon assimilation and structural integrity, possibly mediated by ethylene and pectin-modifying enzymes. The molecular mechanisms linking early light environment to fruit texture likely involve the regulation of cell wall-modifying enzymes. Postharvest studies on tomato have demonstrated that LED radiation significantly affects texture parameters through modulation of genes involved in cell wall metabolism, including pectin methylesterase (PME), polygalacturonase (PG), and β-galactosidase (β-Gal) (Pan et al., 2023). These enzymes govern the disassembly of cell wall components during fruit ripening, and their expression patterns can be influenced by light spectrum. In our study, the enhanced firmness observed in specific treatments may result from light-mediated suppression of these cell wall-degrading enzymes during fruit development, leading to greater structural integrity at harvest. The cultivar-specific responses in textural properties further suggest that genetic factors determine the sensitivity of cell wall metabolism to light signals.

### 4.3 Vitamin C and titratable acid

The significant increase in vitamin C content observed in ‘Green Gem’ under A3 treatment, contrasted with the absence of response in ‘Young White Lady’, reveals cultivar-specific regulation of ascorbate metabolism by light spectrum. Vitamin C is a major nutritional quality attribute in fruits and vegetables, and its biosynthesis is known to be influenced by light through transcriptional regulation of biosynthetic genes and substrate availability. Recent studies have shown that the light signaling transcription factor HY5 can directly activate CDF3 expression, which subsequently binds to the GGP2 promoter to promote ascorbic acid accumulation (Huang et al., 2025). The differential response between ‘Green Gem’ and ‘Young White Lady’ may reflect variation in the expression of GDP-L-galactose phosphorylase or other key enzymes in the ascorbate biosynthesis pathway, or differences in the sensitivity of these enzymes to light-mediated signaling.

The consistent and significant suppression of titratable acidity across almost all LED treatments in both cultivars represents one of the most robust findings of this study. The reduction in titratable acid content, coupled with stable or enhanced sugar levels, resulted in markedly improved sugar-acid ratios—a critical determinant of melon sensory quality and consumer preference (Zhang et al., 2025). Similar phenomena have been observed in a postharvest study on pitaya, red light delayed the increase in TA and the decline in TSS-TA ratio, while blue light significantly reduced the upward trend in TA (Wu et al., 2020). The correlation analysis revealed that titratable acidity increased monotonically with higher red-to-blue ratio (ρ = 0.579, *p <* 0.01), while the sugar-acid ratio decreased significantly (ρ = -0.465, *p <* 0.01), further confirming that spectral composition strongly influences organic acid metabolism. A tomato study has similarly found that 100% blue light treatment can affect secondary metabolite accumulation, though the persistent effects of seedling-stage treatments may diminish after transplanting (Lee et al., 2025). The carry-over effect from seedling stage to fruit maturity suggested that early-light environment programs the metabolic machinery that will later determine acid accumulation during fruit ripening. Genetic studies on sugar and acid accumulation in melon have identified multiple QTL clusters on chromosomes 4, 5, and 7 associated with sugar and organic acid composition (Argyris et al., 2017).

### 4.4 Seasonal variation and environmental integration

The pronounced seasonal variation observed across all sensory and physicochemical attributes, with S3 and S4 exhibiting superior sweetness, aroma, and overall acceptability while S1 showed elevated bitterness and astringency, underscores the dominant influence of growing environment on fruit quality. This variation reflects the complex integration of light, temperature, humidity, and other environmental factors during fruit development and ripening (Vallone et al., 2013). As shown in **Figure S2**, solar radiation levels during the seedling stages (weeks 1–5) did not differ significantly across the four seasons, ranging from 7 to 13 MJ/m². However, the post-transplanting growth conditions in S3 and S4 were more favorable, characterized by higher light intensity and moderately increased temperatures after transplanting (week 6) in the early spring environment of eastern China. The greenhouse experienced severe low-light stress from January 23 to February 27 (**Figure S3**). These conditions positively contributed to the development and quality of oriental melons. In contrast, the use of LED lighting at an intensity of 18 W during the seedling stage proved unsuitable for the winter conditions of S1 and S2. For optimal results, an intensity of 48–60 W was most effective for oriental melons, and supplementary lighting should be maintained after transplanting.

### 4.5 Principal component analysis and quality trade-offs

Principal component analysis revealed a fundamental trade-off between texture properties and sensory attributes along PC1 (accounting for 25.77% of total variance). This inverse relationship between structural integrity (firmness, adhesiveness, chewiness) and desirable flavor traits (sweetness, aroma, overall acceptability) poses a key challenge for breeding and cultivation management. The biological basis of this trade-off likely involves multiple interconnected mechanisms operating at physiological and molecular levels. Fruits that maintain firmer texture through reduced cell wall degradation may exhibit lower sugar accumulation or volatile production, as these processes are typically coupled with ripening-associated softening (Pan et al., 2023). One plausible explanation lies in the competition for carbon assimilates between structural components and primary metabolism. Fruits that maintain firmer texture through enhanced cell wall deposition or reduced degradation may allocate a greater proportion of photosynthates to structural polysaccharides (cellulose, hemicellulose, pectin) at the expense of soluble sugar accumulation. This source-sink balance is critically regulated during fruit development, with carbon partitioning between cell wall synthesis and sugar storage representing a potential point of metabolic trade-off (Falchi et al., 2020). Cell wall metabolism and sugar signaling are interconnected, as softening involves enzymatic disassembly (e.g., PG, PL, β-galactosidase) that releases sugars from polysaccharides, impacting both texture and flavor. Studies in tomato have demonstrated that suppressing cell wall-modifying enzymes can delay softening but may also affect sugar mobilization and flavor development. Furthermore, sugar signaling pathways interact with hormone networks that regulate both softening and flavor biosynthesis (Wang and Seymour, 2022). Ethylene and ABA coordinately regulate ripening processes, often linking softening and flavor changes. However, specific regulators like SlLOB1 suggest potential to decouple texture-flavor trade-offs (Shi et al., 2021).

### 4.6 Mechanistic framework and future directions

Integrating our findings with those of previous studies on oriental melon, we propose a mechanistic framework for the carry-over effects of seedling-stage LED lighting on melon fruit quality. Seedling photoreceptors (phytochromes, cryptochromes/phototropins) perceive light signals and activate transcription factors such as *CmHY5* and *CmWRKY28*, which may establish persistent epigenetic or transcriptional programs that shape metabolism, cell wall architecture, and hormone balance through development, ultimately determining harvest quality (Guan et al., 2026). Cultivar-specific responses likely reflect differences in photoreceptor sensitivity, transcription factor activity, or epigenetic regulation. Given the established roles of *CmHY5* and *CmWRKY28* in red-light-mediated sucrose accumulation, future studies should examine their expression or allelic variation between ‘Green Gem’ and ‘Young White Lady’ and their correlation with fruit sugar content under different light treatments. From a practical standpoint, targeted spectral management during the seedling stage represents a viable strategy for modulating specific quality attributes in melon. For instance, the application of R: B = 5:1 at 18 W in ‘Green Gem’ effectively reduced bitterness while enhancing sweetness, demonstrating the potential of tailored light recipes for precision quality improvement.

Observed seasonal variation underscores the need for multi-environment trials. Optimal light recipes may require adjustment based on predicted growing conditions, supporting the development of predictive models that integrate light treatment parameters with environmental data. During winter cultivation, light intensity appears to play a more critical role than spectral composition in determining outcomes. Full-spectrum LEDs, owing to their broader applicability, offer a cost-effective solution by minimizing the need for frequent spectral adjustments or lamp replacements. This approach alleviates practical concerns associated with tailoring spectra to specific varieties.

## 5 Conclusions

This study demonstrates that LED supplementary lighting during the seedling stage programs the texture and eating quality of oriental melon at maturity, with effects critically dependent on cultivar-specific responses to light spectrum and intensity. For the cultivar ‘Green Gem’, quality was predominantly governed by spectral composition, with a R:B ratio of 5:1 (A2) optimizing sensory perception by significantly reducing bitterness and enhancing sweetness. In contrast, for ‘Young White Lady’, quality was primarily driven by light intensity, with full-spectrum white light at 48 W (B5) most effectively promoting TSS, sugar-acid ratio, and overall acceptability. A universal trade-off was identified between textural firmness and desirable flavor attributes, forming the primary axis of quality variation. Texture qualities such as cohesiveness and chewiness showed strong positive correlations with red-orange and blue-violet effective irradiance, whereas a high R:B ratio was a dominant factor inducing titratable acid accumulation, thereby compromising flavor by lowering the sugar-acid ratio. Future research should focus on elucidating these underlying molecular mechanisms through transcriptomic and metabolomic approaches, and to refine dynamic light recipes that account for cultivar genetics and seasonal environmental interactions. This work establishes seedling-stage spectral management as a viable strategy for the precise and sustainable quality control of high-value horticultural crops.

## Data Availability

The original contributions presented in the study are included in the article/**Supplementary Material**. Further inquiries can be directed to the corresponding author.

## References

[B1] Appolloni E. Orsini F. Pennisi G. Gabarrell Durany X. Paucek I. Gianquinto G. (2021). Supplemental LED lighting effectively enhances the yield and quality of greenhouse truss tomato production: results of a meta-analysis. Front. Plant Sci. 12, 596927. doi: 10.3389/fpls.2021.596927. PMID: 33995427 PMC8118716

[B2] Argyris J. M. Díaz A. Ruggieri V. Fernández M. Jahrmann T. Gibon Y. . (2017). QTL analyses in multiple populations employed for the fine mapping and identification of candidate genes at a locus affecting sugar accumulation in melon (Cucumis melo L.). Front. Plant Sci. 8, 1679. doi: 10.3389/fpls.2017.01679. PMID: 29018473 PMC5623194

[B3] Bianchi T. Guerrero L. Gratacós-Cubarsí M. Claret A. Argyris J. Garcia-Mas J. . (2016). Textural properties of different melon (Cucumis melo L.) fruit types: sensory and physical-chemical evaluation. Sci. Hortic. 201, 46–56. doi: 10.1016/j.scienta.2016.01.028. PMID: 41869561

[B4] Brini F. Mseddi K. Brestic M. Landi M. (2022). Hormone-mediated plant responses to light quality and quantity. Environ. Exp. Bot. 202, 105026. doi: 10.1016/j.envexpbot.2022.105026. PMID: 41869561

[B5] Chen S. Kerstens T. Zepeda B. Ouzounis T. Olschowski S. Marcelis L. . (2024). Additional far-red increases fruit yield of greenhouse sweet pepper mainly through enhancing plant source strength. Sci. Hortic. 338, 113787. doi: 10.1016/j.scienta.2024.113787. PMID: 41869561

[B6] Chen X. Wang L. Li T. Yang Q. Guo W. (2019). Sugar accumulation and growth of lettuce exposed to different lighting modes of red and blue LED light. Sci. Rep. 9, 6926. doi: 10.1038/s41598-019-43498-8. PMID: 31061448 PMC6502839

[B7] de Freitas S. Q. Negreiros A. M. P. Nunes G. H. D. Cavalcante A. L. A. Santos F. J. Q. Viana D. M. . (2024). Genetic diversity and response of melon accessions to Monosporascus cannonballus. J. Phytopathol. 172, e13384. doi: 10.1111/jph.13384. PMID: 41858021

[B8] Falchi R. Bonghi C. Drincovich M. F. Famiani F. Lara M. V. Walker R. P. . (2020). Sugar metabolism in stone fruit: source-sink relationships and environmental and agronomical effects. Front. Plant Sci. 11, 573982. doi: 10.3389/fpls.2020.573982. PMID: 33281843 PMC7691294

[B9] Farcuh M. Copes B. Le-Navenec G. Marroquin J. Jaunet T. Chi-Ham C. . (2020). Texture diversity in melon (Cucumis melo L.): sensory and physical assessments. Postharvest Biol. Technol. 159. doi: 10.1016/j.postharvbio.2019.111024. PMID: 41869561

[B10] Gao W. She F. Sun Y. Han B. Wang X. Xu G. (2023). Transcriptome analysis reveals the genes related to water-melon fruit expansion under low-light stress. Plants 12, 935. doi: 10.3390/plants12040935. PMID: 36840282 PMC9958833

[B11] Grabowski J. A. Truong V. D. Daubert C. R. (2008). Nutritional and rheological characterization of spray dried sweetpotato powder. LWT Food Sci. Technol. 41, 206–216. doi: 10.1016/j.lwt.2007.02.019. PMID: 41869561

[B12] Guan J. Gao G. Yang F. Wang J. Pan J. Liu T. . (2026). CmHY5 and CmWRKY28 regulate sucrose accumulation in oriental melon under supplemental red light. J. Integr. Plant Biol. 68, 760–776. doi: 10.1111/jipb.70096. PMID: 41299199

[B13] Guan J. Liang X. Gao G. Yang F. Qi H. (2024). The interaction between CmPIF8 and CmACO1 under postharvest red light treatment might affect fruit ripening and sucrose accumulation in oriental melon fruit. Postharvest Biol. Technol. 209, 112717. doi: 10.1016/j.postharvbio.2023.112717. PMID: 41869561

[B14] Han X. Zhang Y. Lou Z. Li J. Wang Z. Gao C. . (2023). Time series single-cell transcriptional atlases reveal cell fate differentiation driven by light in Arabidopsis seedlings. Nat. Plants 9, 2095–2109. doi: 10.1038/s41477-023-01544-4. PMID: 37903986 PMC10724060

[B15] Hossain M. M. Shibasaki Y. Goto F. (2025). Enhancement of growth and quality of winter watermelon using LED supplementary lighting. Horticulturae 11, 262. doi: 10.3390/horticulturae11030262. PMID: 41725453

[B16] Huang Q. Yan Y. Zhang X. Cao X. Ludlow R. Lu M. . (2025). Cycling Dof Factor 3 mediates light-dependent ascorbate biosynthesis by activating GDP-L-galactose phosphorylase in Rosa roxburghii fruit. Plant Physiol. 197, kiaf014. doi: 10.1093/plphys/kiaf014. PMID: 39797913

[B17] ISO (2003). 2173:2003 Fruit and vegetable products-Determination of soluble solids—Refractometric method (Geneva: International Organization for Standardization).

[B18] ISO (2012). 8586:2012 Sensory analysis-General guidelines for the selection, training and monitoring of selected assessors and expert sensory assessors (Geneva: International Organization for Standardization).

[B19] Jadidi M. Mumivand H. Nia A. E. Shayganfar A. Maggi F. (2023). UV-A and UV-B combined with photosynthetically active radiation change plant growth, antioxidant capacity and essential oil composition of Pelargonium graveolens. BMC Plant Biol. 23, 555. doi: 10.1186/s12870-023-04556-6. PMID: 37946108 PMC10636913

[B20] Kim H. A. Shin A.-Y. Lee M.-S. Lee H.-J. Lee H.-R. Ahn J. . (2016). De novo transcriptome analysis of Cucumis melo L. var. makuwa. Mol. Cells 39, 141–148. doi: 10.14348/molcells.2016.2264. PMID: 26743902 PMC4757802

[B21] Lázaro A. de Lorenzo C. (2015). Texture analysis in melon landraces through instrumental and sensory methods. Int. J. Food Prop. 18, 1575–1583. doi: 10.1080/10942912.2014.923441. PMID: 41858497

[B22] Lee C. Nagila A. Harvey J. T. Choi S. Leskovar D. I. (2025). Humic substances and LED lighting improved tomato transplant growth, with limited carryover effects on fruit yield and quality. HortScience 60, 476–486. doi: 10.21273/hortsci18343-24

[B23] Li Z. Chen Q. Xin Y. Mei Z. Gao A. Liu W. . (2021). Analyses of the photosynthetic characteristics, chloroplast ultrastructure, and transcriptome of apple (Malus domestica) grown under red and blue lights. BMC Plant Biol. 21, 483. doi: 10.1186/s12870-021-03262-5. PMID: 34686132 PMC8539889

[B24] Li X. Lu W. Hu G. Wang X. C. Zhang Y. Sun G. X. . (2016). Effects of light-emitting diode supplementary lighting on the winter growth of greenhouse plants in the Yangtze River Delta of China. Bot. Stud. 57, 2. doi: 10.1186/s40529-015-0117-3. PMID: 28597414 PMC5430560

[B25] Luo F. Huang Y. Sun Y. Guan J. Li M. Liu T. . (2024). Transcription factor CmWRKY13 regulates cucurbitacin B biosynthesis leading to bitterness in oriental melon fruit (Cucumis melo var. Makuwa Makino). J. Agric. Food. Chem. 72, 24697–24710. doi: 10.1021/acs.jafc.4c04608. PMID: 39460931

[B26] Ma X. Wang Y. Li X. Liu Y. Luo H. Shang W. . (2025). Integrative analysis of different low-light-tolerant watermelon lines in response to low-light stress. BMC Plant Biol. 25, 1107. doi: 10.1186/s12870-025-07180-8. PMID: 40841994 PMC12369212

[B27] Mei Z. Li Z. Lu X. Zhang S. Liu W. Zou Q. . (2023). Supplementation of natural light duration promotes accumulation of sugar and anthocyanins in apple (Malus domestica Borkh.) fruit. Environ. Exp. Bot. 205, 105133. doi: 10.1016/j.envexpbot.2022.105133. PMID: 41869561

[B28] Miao Y. Chen Q. Qu M. Gao L. Hou L. (2019). Blue light alleviates ‘red light syndrome’ by regulating chloroplast ultrastructure, photosynthetic traits and nutrient accumulation in cucumber plants. Sci. Hortic. 257, 108680. doi: 10.1016/j.scienta.2019.108680. PMID: 41869561

[B29] Moreno-Cuenca L. Navas-Guzmán F. Doppler L. Moreno I. (2025). Evaluation of the partition of global solar radiation into UVA, PAR, and NIR components in a rural environment. Remote Sens. 17, 3439. doi: 10.3390/rs17203439. PMID: 41725453

[B30] Pan H. Li M. Liu T. Qi H. (2023). Multi-microscopy techniques combined with FT-IR spectroscopy reveals the histological and biochemical causes leading to fruit texture difference in oriental melon (Cucumis melo var. Makuwa Makino). Food Chem. 402, 134229. doi: 10.1016/j.foodchem.2022.134229. PMID: 36182778

[B31] Pan H. Sun Y. Qiao M. Qi H. (2022). Beta-galactosidase gene family genome-wide identification and expression analysis of members related to fruit softening in melon (Cucumis melo L.). BMC Genomics 23, 795. doi: 10.1186/s12864-022-09006-5. PMID: 36460944 PMC9716742

[B32] Paponov M. Kechasov D. Lacek J. Verheul M. J. Paponov I. A. (2019). Supplemental light-emitting diode inter-lighting increases tomato fruit growth through enhanced photosynthetic light use efficiency and modulated root activity. Front. Plant Sci. 10, 1656. doi: 10.3389/fpls.2019.01656. PMID: 31998343 PMC6965351

[B33] Shi Y. Vrebalov J. Zheng H. Xu Y. Yin X. Liu W. . (2021). A tomato LATERAL ORGAN BOUNDARIES transcription factor, SlLOB1, predominantly regulates cell wall and softening components of ripening. PNAS 118, e2102486118. doi: 10.1073/pnas.2102486118. PMID: 34380735 PMC8379924

[B34] Shi Y. Wang B. Shui D. Cao L. Wang C. Yang T. . (2015). Effect of 1-methylcyclopropene on shelf life, visual quality and nutritional quality of netted melon. Food Sci. Technol. Int. 21, 175–187. doi: 10.1177/1082013214520786. PMID: 24495994

[B35] Shin A.-Y. Koo N. Kim S. Sim Y. M. Choi D. Kim Y.-M. . (2019). Draft genome sequences of two oriental melons, Cucumis melo L. var. makuwa. Sci. Data 6, 220. doi: 10.1038/s41597-019-0244-x. PMID: 31641135 PMC6805853

[B36] Silveira A. C. Aguayo E. Artés F. (2013). Shelf-life and quality attributes in fresh-cut Galia melon combined with fruit juices. LWT Food Sci. Technol. 50, 343–348. doi: 10.1016/j.lwt.2012.04.010. PMID: 41869561

[B37] Tzuri G. Dafna A. Itzhaki B. Halperin I. Oren E. Isaacson T. . (2025). Meta genetic analysis of melon sweetness. Theor. Appl. Genet. 138, 68. doi: 10.1007/s00122-025-04863-6. PMID: 40067361 PMC11897113

[B38] Vallone S. Sivertsen H. Anthon G. E. Barrett D. M. Mitcham E. J. Ebeler S. E. . (2013). An integrated approach for flavour quality evaluation in muskmelon (Cucumis melo L. reticulatus group) during ripening. Food Chem. 139, 171–183. doi: 10.1016/j.foodchem.2012.12.042. PMID: 23561094

[B39] Wang W. Liu X. Cheng C. Xie X. Li L. Bai J. . (2022). Effects of salt and drought stresses and light quality on vitamin C content and expression of synthetic genes in kiwifruit leaves (in Chinese with English abstract). J. Fruit Sci. 39, 203–210. doi: 10.13925/j.cnki.gsxb.20210392

[B40] Wang D. Seymour G. B. (2022). Molecular and biochemical basis of softening in tomato. Mol. Horticulture 2, 5. doi: 10.1186/s43897-022-00026-z. PMID: 37789493 PMC10515243

[B41] Wang L. Wu X. Yu B. Han Y. Zhang Y. Xu D. . (2023). Red light activates H₂O₂ signal involved in powdery mildew resistance in oriental melon seedlings. Environ. Exp. Bot. 215, 105508. doi: 10.1016/j.envexpbot.2023.105508. PMID: 41869561

[B42] Wang J. Yang Z. Gao G. Qi H. (2026). CmMYB113 mediated red light-induced ethylene biosynthesis to modulate the ester aroma in oriental melon. Int. J. Biol. Macromol. 344, 150491. doi: 10.1016/j.ijbiomac.2026.150491. PMID: 41579997

[B43] Wei H. Wang M. Jeong B. R. (2020). Effect of supplementary lighting duration on growth and activity of antioxidant enzymes in grafted watermelon seedlings. Agronomy 10, 337. doi: 10.3390/agronomy10030337. PMID: 41725453

[B44] Wu Q. Zhou Y. Zhang Z. Li T. Jiang Y. Gao H. . (2020). Effect of blue light on primary metabolite and volatile compound profiling in the peel of red pitaya. Postharvest Biol. Technol. 160, 111059. doi: 10.1016/j.postharvbio.2019.111059. PMID: 41869561

[B45] Xu X. Wu S. Chen K. Zhang H. Zhou S. Lv Z. . (2023). Comprehensive evaluation of raw eating quality in 81 sweet potato (Ipomoea batatas (L.) Lam) varieties. Foods 12, 261. doi: 10.3390/foods12020261. PMID: 36673353 PMC9858325

[B46] Yamaguchi M. Hughes D. L. Yabumoto K. Jennings W. G. (1977). Quality of cantaloupe muskmelons: variability and attributes. Sci. Hortic. 6, 59–70. doi: 10.1016/0304-4238(77)90079-6

[B47] Yan W. K. Zhang Y. Q. Zhang Y. T. Cheng R. F. Zhang Y. Yang Q. C. . (2018). Effects of supplementary artificial light on growth of the tomato (Solanum lycopersicum) in a Chinese solar greenhouse. Hortic. J. 87, 516–523. doi: 10.2503/hortj.OKD-165

[B48] Yang L. Chen J. Sun X. Li J. Chen N. (2019). Inhibition of sucrose and galactosyl-sucrose oligosaccharide metabolism in leaves and fruits of melon (Cucumis melo L.) under low light stress. Sci. Hortic. 244, 343–351. doi: 10.1016/j.scienta.2018.09.001. PMID: 41869561

[B49] Yang S. Gong C. Tian Q. Bu Y. Wang Z. Guo W. (2025). Maturation and cultivar effect on optical properties and qualities of melon tissues: optical-based quality evaluation. J. Food Compos. Anal. 148, 108104. doi: 10.1016/j.jfca.2025.108104. PMID: 41869561

[B50] Yu Y. F. Kleuter M. Dinani S. T. Trindade L. M. Goot A. J. V. (2023). The role of plant age and leaf position on protein extraction and phenolic compounds removal from tomato (Solanum lycopersicum) leaves using food-grade solvents. Food Chem. 406, 10. doi: 10.1016/j.foodchem.2022.135072. PMID: 36470086

[B51] Zeng X. Ye L. Zhang R. Wang P. (2025). GLK2, a GOLDEN2-LIKE transcription factor, directly regulates anthocyanin accumulation by binding with promoters of key anthocyanin biosynthetic genes in Arabidopsis. Plant Cell Environ. 48, 7055–7071. doi: 10.1111/pce.15675. PMID: 40518742

[B52] Zhang J. Bai X. Wu J. Zhou B. (2025). Nondestructive detection method for soluble solids content and titratable acidity content in pepino melons based on Vis/NIR spectroscopy and dual-attention enhanced 1D-CNN. J. Food Compos. Anal. 148, 108232. doi: 10.1016/j.jfca.2025.108232. PMID: 41869561

[B53] Zhang S. Zhang L. Zou H. Qiu L. Zheng Y. Yang D. . (2021). Effects of light on secondary metabolite biosynthesis in medicinal plants. Front. Plant Sci. 12, 781236. doi: 10.3389/fpls.2021.781236. PMID: 34956277 PMC8702564

[B54] Zhao S. Li X. Kang Y. Lin Y. Wu Y. Yang Z. (2024). Photomorphogenesis and photosynthetic trait changes in melon seedlings responding to red and blue light. Horticulturae 10, 961. doi: 10.3390/horticulturae10090961. PMID: 41725453

