# Supplementary material for: Effects of seedling-stage LED supplementary lighting on the eating quality and textural properties of oriental melon (Cucumis melo L. var. makuwa Makino) fruits at maturity
Source: Front Plant Sci. 2026 Apr 15;17:1818870. doi: 10.3389/fpls.2026.1818870 (PMC13126549; doi:10.3389/fpls.2026.1818870)
Supplement: Supplementary file 1 [file SupplementaryFile1.docx]

Supplementary Material

# Supplementary Figures and Tables

#
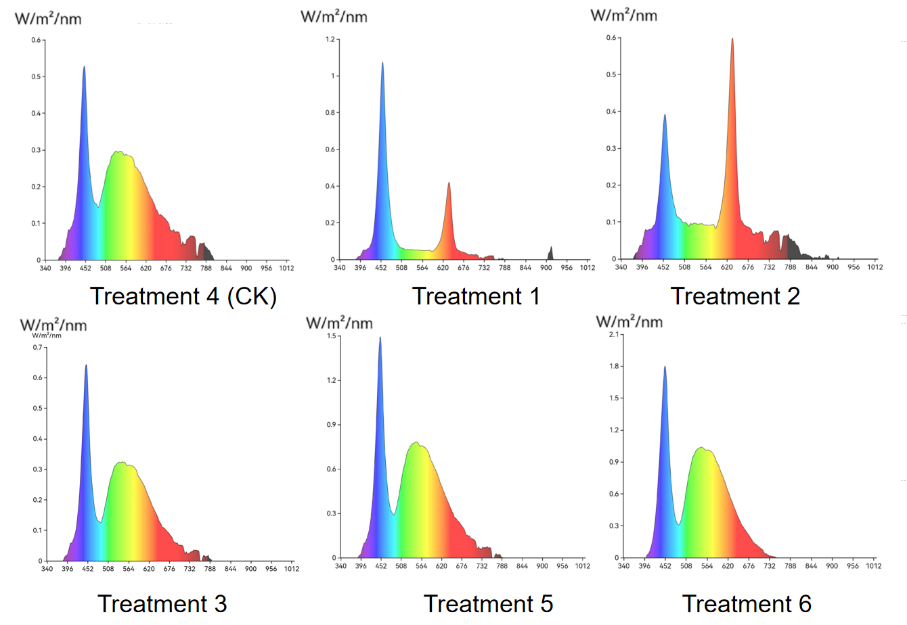


# Supplementary Figure 1. Spectral characteristics in six LED supplementary lighting treatments

#
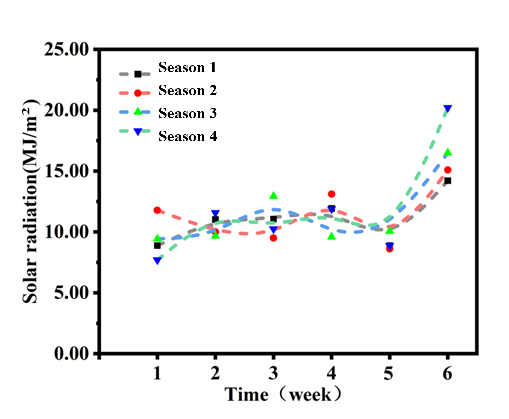


# Supplementary Figure 2. Weekly dynamics of solar radiation in the greenhouse across four seasons


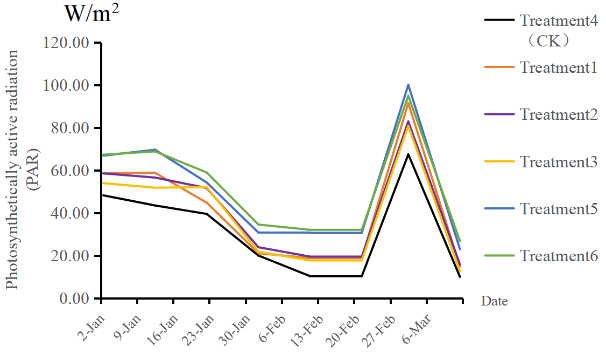

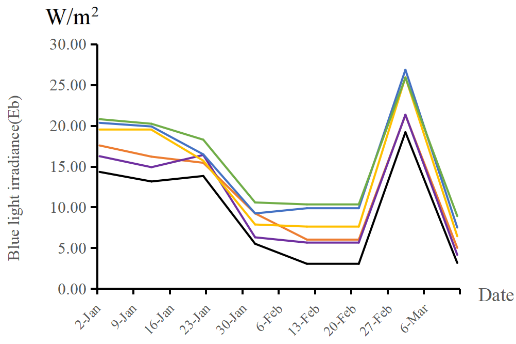


| (**a**) | (**b**) |
| --- | --- |


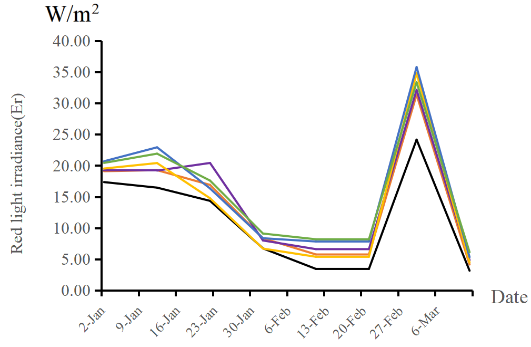

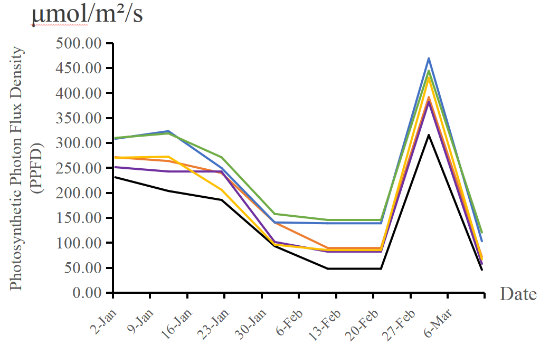


| (**c**) | (**d)** |
| --- | --- |

**Supplementary Figure 3.** Effects of six LED supplementary lighting on greenhouse ligting conditions. (**a**) PAR; (**b**)Eb;(**c**) Er ;(**d**) PPFD
